# Imaginative Culture and the Enriched Nature of Positive Experience

**DOI:** 10.3389/fpsyg.2022.831118

**Published:** 2022-02-28

**Authors:** Nathaniel F. Barrett

**Affiliations:** Institute for Culture and Society, University of Navarra, Pamplona, Spain

**Keywords:** imaginative culture, enjoyment, affect, dynamics of consciousness, John Dewey, Alfred North Whitehead

## Abstract

To understand the evolution of imaginative culture, we need to understand its unique affective power. The purpose of this article is to explain our enjoyment of imaginative culture from the standpoint of a distinctive theoretical approach to understanding affect in terms of the dynamic and energetic features of consciousness. This approach builds upon John Dewey’s view of enjoyment as the enrichment of experience, adding perspectives from studies of the dynamics of consciousness and from ecological psychology. Its main thesis is that positive affect is determined by the causal enrichment of experience, which is defined as the differentiated-ness of conscious states. This approach suggests that the affective power of imaginative culture lies in the way it affords experiences of enriched meaning, as exemplified by our enjoyment of highly nuanced emotion in music.

## Introduction

“Human experience in the large, in its coarse and conspicuous features, has for one of its most striking features preoccupation with direct enjoyment, feasting and festivities; ornamentation, dance, song, dramatic pantomime, telling yarns and enacting stories. In comparison with intellectual and moral endeavor, this trait of experience has hardly received the attention from philosophers that it demands. Even philosophers who have conceived that pleasure is the sole motive of man and the attainment of happiness his whole aim, have given a curiously sober, drab, account of the working of pleasure and the search for happiness. Consider the utilitarians how they toiled, spun and wove, but who never saw man arrayed in joy as the lilies of the field. Happiness was to them a matter of calculation and effort, of industry guided by mathematical book-keeping. The history of man shows however that man takes his enjoyment neat, and at as short range as possible” Dewey (1958, p. 78).

Surely one of the most prominent and distinctive traits of the human species is our insatiable appetite for *imaginative culture*. Broadly speaking, imaginative culture includes all the arts, many varieties of entertainment, and a good part of religion. It is found in every human society and in every era and comes in an endless variety of forms, from cave paintings to video games. More precisely, for the purposes of this article, imaginative culture refers to all the different ways in which we seek to enrich our experience through the use of specially prepared media. In this article I shall be focusing on the arts, especially music, which I take to be representative of imaginative culture in the following respect: Whatever other uses it might have, at the heart of all imaginative culture is the cultivation of enriched experiences that cannot be had except through special means—song and dance, paintings, costume, and ritual—in short, all the specially organized materials and activities that make up various “technologies of the imagination.”

On this view, imaginative culture is understood primarily as a source of enjoyment. It follows that our insatiable appetite for this culture is driven by a “preoccupation with direct enjoyment,” as suggested by Dewey in the passage above. This view can be applied to the emergence of new forms of imaginative culture in the present as well as its origin in our distant past. In short, to understand the evolution of imaginative culture, we must understand how it works as a source of enjoyment and we must understand why this form of enjoyment seems to be unique to our species.

From this standpoint, I contend that Dewey’s critique still holds today. We do not have a scientific theory that adequately explains our enjoyment of imaginative culture. But the problem is much deeper than this, as we do not have a theory that explains our experience of any kind of enjoyment or suffering. Moreover, I suggest that the main obstacle is not the limited state of present knowledge, but rather a widespread failure to come to grips with the special challenges presented by the affective nature of experience. Most scientific treatments of pleasure only explain why we enjoy *this* or *that* (e.g., see Bloom, 2011); they do not explain what enjoyment *is*.

The main purpose of this article is not to criticize other theories, however. Rather, the purpose is to present a theory of affect that can help us to make progress on questions about enjoyment that are too often set aside as beyond the reach of scientific inquiry. In particular, I want to show that our seemingly species-unique enjoyment of imaginative culture is a critical piece of a much bigger puzzle that I call the “problem of affect.” Thus, to understand our enjoyment of imaginative culture, we have to understand how it relates to other kinds of pleasure and enjoyment. At the same time, I want to show that the specially enriched nature of our experience of imaginative culture provides an important clue to understanding the nature of affect in general.

As I explain in the next section (see “The Problem of Affect”), at the heart of the problem of affect is the peculiarly elusive and yet unmistakable feeling of affective valence or hedonic tone. The rest of the article is devoted to the articulation of a theoretical framework that I believe is able to face up to the special challenges of this problem.

First, I present the general outlines of a theoretical approach (see “The Enrichment Approach”) that builds upon Dewey’s understanding of aesthetic enjoyment as “the clarified and intensified development of traits that belong to every normally complete experience” Dewey (1980, p. 46). I call this the “enrichment approach” to the understanding of affect. In brief, the enrichment approach seeks to understand enjoyment as the enrichment of experience as a whole rather than the addition of a special ingredient.

Next, to develop this approach into a more comprehensive theory of affect, I propose to define affect as the causal enrichment of experience (see “The Causal Enrichment of Experience”). My central thesis is that changes of affective tone are related to the *differentiated-ness* of conscious states. Insofar as differentiated-ness is related to energy use, it follows from this thesis that positive affect makes energetic demands that can be met in two ways: through the body’s own mechanisms of affective regulation or through engagement with the ambient arrays of energy on which perceptual experience “feeds.” These two main sources of enrichment correspond to two basic kinds of positive experience, and I suggest that our enjoyment of imaginative culture is a special variety of the latter.

In the final section (see “Imaginative Culture and the Enjoyment of Enriched Meaning”), I suggest that our seemingly species-unique enjoyment of imaginative culture is dependent on our ability to engage with the meanings carried by specially prepared media. As exemplified by our enjoyment of highly nuanced emotion in music, our enjoyment of imaginative culture is distinguished by the experiences of enriched meaning that it affords. I close with the suggestion that we use the enrichment of imaginative culture to explore and transfigure our experiences of the most troubling aspects of life.

## The Problem of Affect

In this section I define the focus of present discussion—that which psychologists refer to as *valence*—and provide a brief exposition of the peculiar challenge that it presents to understanding.

In both psychology and philosophy, *affect* encompasses a diverse range of physiological, behavioral, and psychological phenomena involved in emotions, moods, sensory pleasures, and physical pains (Fox, 2008). Here, we are primarily interested in our experience of affect, and we will be focusing on the affective trait of *valence*. As discussed here, valence is equivalent to pleasantness and hedonic tone. It refers to the pre-reflective, immediately felt, intrinsic value of experience. Valence is widely assumed to be a “fundamental, universal property of human experience,” such that “every person on the planet (barring illness) can tell good from bad, positive from negative, pleasure from displeasure” (Lindquist et al., 2016, p. 1910).

Due to space constraints, the following discussion is largely restricted to the positive side of valence. Although there are important differences between pain and pleasure, the problem of affect to be articulated here applies to both in more or less the same way.

Also, throughout the following discussion, it should be kept in mind that affect includes more than just valence. Most theories of affect include an “energetic” dimension, variously called *arousal*, *excitement*, *intensity*, or *tension* (Barrett and Bliss-Moreau, 2009). Nevertheless, valence has a certain pride of place as the central feature in relation to which all affective phenomena—experiential, behavioral, physiological, and cognitive—are defined as such. In short, while valence is by no means the entirety of affect, it is the linchpin that holds our concept of affect together. It is also by far the most challenging aspect of affect to describe and explain. Indeed, I suggest that our feeling of value is one of those things that, as Augustine famously said of time, we understand only as long as no one asks us to explain them.

When I claim that most scientists have failed to come to grips with the special challenges presented by the affective nature of experience, I am referring especially to the feeling of valence. At first blush, such a sweeping claim is hard to square with the way in which affect has moved to the center of scientific and philosophical discussions of thought and behavior during the past several decades (e.g., Damasio, 1994; Panksepp and Biven, 2012; Colombetti, 2014; Asma and Gabriel, 2019). Still, for a number of reasons, I contend that questions about the nature of affect, and about valence in particular, have become a persistent blind spot even within affective science.

One reason is the fact that affective valence has been successfully operationalized for empirical study. Scientists have methods for eliciting positive and negative states (moods or feelings) and for measuring these states through self-report. Along as these methods are deemed reliable, and as long as a wealth of data can be mined from studies of how these states relate to other measurable effects (e.g., on cognition), it seems that scientists need not enter into questions about *how* we feel valence. Moreover, such questions about, the feeling of affect have long been set outside the boundaries of scientific inquiry as demarcated by the so-called “hard problem” of consciousness (Chalmers, 1996).

The most influential formulations of the problem of consciousness (Nagel, 1974; Levine, 1983; Jackson, 1986; Chalmers, 1996) assert that a complete functional or causal account of mental processes could, in theory, be provided in exclusively non-experiential or “third-person” terms. That is, *if* such an account were to be attained, it would still leave the feelings of consciousness unexplained—this is why the problem of consciousness is “hard.” Or so the argument goes. Whatever its original motivation, this line of argumentation has contributed to an attitude of complacency toward questions about experience. If our feeling of affect has no place in a complete functional or causal understanding of affect, it is a “phenomenal residue” (Crane, 2019) that can be disregarded by scientists. This way of thinking has also contributed to a widespread tendency to conflate affect with other aspects of experience: all feelings can be lumped together and relegated to the other side of an “explanatory gap.”

As a result of this demarcation, scientific explanations of affect typically leave experience aside and focus on one or more of the following: description of functions (e.g., homeostatic regulation), identification of causal mechanisms (e.g., neurochemicals, such as vasopressin and oxytocin), and adaptive-functionalist accounts of how these mechanisms have evolved to support our various species-specific likes and dislikes (e.g., see Bloom, 2011). Behind this explanatory approach is a mechanistic idea of affect, which I suggest is responsible for the current limitations of affective science.

As a representative example, consider Pinker’s (1997, p. 534) famous conjecture that music is “auditory cheesecake, an exquisite confection crafted to tickle the sensitive spots of at least six of our mental faculties”. In its original context, this conjecture about music deliberately echoes earlier remarks about the pleasure of cheesecake: “Cheesecake packs a sensual wallop unlike anything in the natural world because it is a brew of megadoses of agreeable stimuli which we concocted for the express purpose of pressing our pleasure buttons” (p. 525). Phrases like “sensitive spots” and “pleasure buttons” are not meant literally, of course. Nevertheless, I suggest that they are accurate expressions of the thoroughly mechanistic thinking behind most scientific explanations of pleasure and pain.

By “mechanistic” I mean to refer to the way in which all affective responses are understood to be *triggered* by certain kinds of stimuli (or, in the case of interoception, certain bodily states). The critical point is that valence, whether positive or negative, is understood as the product of a mechanism that is activated or “turned on” in such a way that affective responses do not discriminate between stimuli that are functionally equivalent. Importantly, this mechanistic concept of affect covers a wide variety of functions (e.g., homeostatic regulation) and causal mechanisms (e.g., neurochemicals). That is, whatever the functional and causal details turn out to be, any affective response that works this way can be described as a “pleasure button.” Insofar as different nervous systems are “wired” differently, different species may have different pleasure buttons. But for any given species, all stimuli that suffice to activate a pleasure mechanism elicit the same affective response. Thus, according to Pinker, although we did not evolve pleasure buttons specifically for music or cheesecake, the only difference between these artificially crafted pleasures and those for which our pleasure buttons originally evolved is the added “wallop” achieved by combining triggers and boosting their strength. In all other respects, affective responses to different stimuli are essentially the same.

There are good reasons why the mechanistic view of pleasure is so widely assumed. It seems plainly evident that different animals are “wired” differently for pleasure—consider, for instance, how dogs seem to be delighted by smells that we find revolting. Moreover, all behavioral conditioning (and perhaps all learning) seems to depend on the existence of a “hard-wired” set of unconditioned responses that function as pleasure and pain buttons. Through conditioning, any stimulus can be made to push these buttons and thereby trigger an equivalent affective response. These are important facts, and all theories of affect should be able to account for them. Even so, when considered from the standpoint of experience, the mechanistic view of pleasure has major shortcomings.

In particular, when applied to our enjoyment of various kinds of imaginative culture, as well as an enormous variety of other commonly enjoyed activities—socializing and festivity, play and sport, hunting and fishing, etc.—the mechanistic view fails to account for the interactive and highly context-specific nature of our experience. In anticipation of later discussion, I suggest that we gather all of these different varieties of enjoyment into a single broad category of positive experience that is distinguished by the way the singular character of an experience of enjoyment develops through interaction. The point I wish to make is that it is difficult to understand this interactive kind of enjoyment as a “triggered” response.

For example, in the case of music, our enjoyment does not seem to be an all-or-nothing response to certain stimuli. Rather it seems to be an extended experience that builds over time and entails the perception of specific features (e.g., nuances of expression), which, in many cases, are unique to the present performance. To be clear, the problem is not that the mechanist view denies that these features are part of our experience; the problem is that it cannot consider these features as contributions to enjoyment. As explained above, insofar as affective valence is something “triggered” or “turned on,” affect does not discriminate between equivalent classes of stimuli. The distinctive features of a musical performance or other work of art therefore cannot enter into enjoyment except as instances of a general class. Whenever the musical triggers of enjoyment are present to a sufficient degree, enjoyment should automatic and it should be recognizable as a common type. It seems, however, that music and other interactively enjoyed activities are *not* automatically enjoyed, and that the character of our enjoyment is as diverse as all the different objects and activities that we find enjoyable in this way. Enjoyment of this kind seems not to depend on any one feature or quality; rather, it seems to depend on or rather *to consist in* the “overall quality” of our experience. This overall quality has to do with specific features of the object or activity as well as our engagement with these features. Enjoyment of this kind is therefore an interactive achievement, not something triggered.

A second and perhaps even deeper shortcoming of the mechanistic view is the implication that valence is a special ingredient—a distinct quality—that is added to experience. Admittedly, because the feeling of affective valence is so rarely described (because, like time, everyone “knows” what it is), it is difficult to pin this view on particular theories. Nevertheless, I submit that valence is widely treated as if it were a distinct quality that is added to experience, and that the plausibility of the mechanistic approach depends in part on this common assumption.

To be sure, this assumption is not unique to affective science. It is based in our ordinary way of talking about pleasures and pains. The intimate and unmistakable presence of affective feelings lead us to believe that we know them by a distinct quality that clearly marks them as pleasant or painful. As observed by Moore (1968, p. 13), pleasure is like color perception in that the presence of a good feeling is as unmistakable as the yellow of a sunflower: “though pleasure is absolutely indefinable, though pleasure is pleasure and nothing else whatever, yet we feel no difficulty in saying that we are pleased”. And yet, during the last century and a half, philosophers and psychologists who have undertaken careful examinations of our feelings of pleasure and pain have concluded that affective valence cannot be identified with any distinct quality. In the words of Charles Peirce, “it is not the fact that any such common quality…is readily to be recognized” (Sidgwick, 1874; Arnold, 1960; Alston, 1967; Peirce, 1998, p. 190; Frijda, 2009; Labukt, 2012; Aydede, 2014; Katz, 2016).

The most common way of pointing out the lack of any distinctive quality that marks pleasantness or painfulness is to compare different feelings of pleasure and pain. The fact that no such quality “is readily to be recognized” is referred to as the “problem of heterogeneity” (Labukt, 2012; Aydede, 2014). The peculiar elusiveness of affect can also be indicated by an examination of the common phenomenon of *alliesthesia* (Cabanac, 1971): a change in the valence of a stimulus, usually caused by a change of internal state (such as a loss of appetite). Instead of comparing different kinds of pleasure, in cases of alliesthesia, we can consider how a single kind of pleasure changes. For example, consider how the pleasantness of chocolate or some other pleasing food changes as a result of binge eating (see Small et al., 2001). What constitutes the difference between pleasant chocolate taste and unpleasant chocolate taste? It is hard to say, and that is the point: we cannot say how the valence of experience changes even when we know for sure that it does. Valence is not marked by any single, discrete quality that we can isolate within experience, not even a vague, diffuse quality similar to ambient temperature, noise, or light. And yet, we have no problem detecting changes of valence and reporting them to others; indeed, we do it all the time.

The absence of any distinct affective quality is at heart of the problem of affect. In brief, this problem can be summarized as follows: *Despite its unmistakable presence and its fundamental and pervasive role in thought and behavior, affect is extremely difficult to describe and analyze because it has no distinctive quality of its own*.

Although the problem of affect is evidently a problem of experience, it is curiously absent from most formulations of the problem of consciousness. This blind spot is likely the result of a pervasive “sensory bias” (Frijda, 2009) in modern thought about consciousness. According to the philosopher (Crane, 2019, p. 94) “over the course of the [last] century, consciousness in the analytic tradition became conceived of as a primarily sensory phenomenon, with the sensory element itself conceived of as something inexpressible, indefinable, inefficacious and separable from the rest of mental life”. This way of thinking about consciousness has been sharply criticized (e.g., Dennett, 1988), but as long as the underlying preoccupation with the qualitative aspects of experience holds sway, philosophers and scientists are prone to overlook the distinctive challenge presented by our feeling of affect. When we feel the blues we do not feel anything like the color blue.

In the philosophical literature that has examined the problem of affect most directly, it remains unresolved (see Katz, 2016). Probably the best indication of the kind of solution we are seeking comes from the “adverbial” view defended by philosopher Murat Aydede (2014, 2018). Aydede (2018, p. 253) argues that we should think of affect not as a distinct feature but a *way* of feeling that modifies the various qualitative contents of experience. For instance, in the case of sensory pleasures like the sweet taste of strawberry, “we need to distinguish the phenomenology of sensations from the phenomenology of pleasantness that *modifies* or qualifies these sensations”. To show what he means, Aydede uses the following analogy: the tempo of a dance can change in a way that modifies our experience of the dance without changing its distinctive form—fast or slow, a tango is still a tango. Aydede is suggesting that something similar happens when the pleasantness of chocolate changes while the taste remains more or less the same.

Helpful as Aydede’s analogy may be, it falls short in two key respects. First, an adequate concept of affective modification must be applicable across *all* sensory modes. Second, an adequate concept of affective modification must somehow illuminate the special connection between affect and value. While changing the tempo of a dance *might* increase our enjoyment, there is no inherent connection between tempo and pleasure. What kind of modification could account for the way in which affective feelings are, in Aydede’s (2018, p. 246) words, “inherently motivating and intrinsically good or bad”?

## The Enrichment Approach

The theory to be presented in the remaining sections of this article is an elaboration of what I call the “enrichment approach” to affect. The basic idea behind this approach is that a good feeling is not a special ingredient added to experience but rather a heightening, intensification, or *enrichment* of experience as a whole. In the words of John Dewey, enjoyment is “the clarified and intensified development of traits that belong to every normally complete experience” Dewey (1980, p. 46). This is why, when we have an unmistakably good feeling, there is no special quality in experience that shows up as a mark of goodness. Rather, you might say that what we feel is an improvement in the activity of experiencing itself.

As I will indicate momentarily, the enrichment approach needs to be significantly revised and expanded before it can be elaborated into a comprehensive theory of affect. But even as an initial orientation toward the problem of affect, the enrichment approach has important implications. In keeping with the adverbial view, it implies that affect is marked by a change in our way of experiencing rather than the addition of a special ingredient. It also implies that affect is integral to experience, so that there can be no such thing as a totally affectless experience. And it implies that consciousness is not an all-or-nothing phenomenon—it can be enriched or impoverished. Finally, it suggests that positive valence consists in the overall richness of experience rather than a distinct quality or feature. But what exactly is meant by the “richness of experience” needs clarification.

The enrichment approach has many precedents stretching back as far as Aristotle and Plato. In contemporary psychology, the most well-known representatives of this approach are Csikszentmihalyi’s (1990) “flow” theory of optimal experience and Fredrickson’s (1998) “broaden-and-build” theory of positive emotion. As a concept of affect, however, enrichment faces serious obstacles, as it seems that no matter how we define affect in terms of richness, we cannot account for certain basic kinds of positive and negative feeling.

In particular, the enrichment approach does not seem able to explain the kinds of reflexive, “button-like” affective responses that lend so much support to the mechanistic view examined above. Indeed, at first glance, it seems as if the enrichment view and the mechanistic view are not competitors but rather complementary approaches to two fundamentally different kinds of positive experience. For even if we grant that enrichment theories are a good fit for all the various kinds of interactive enjoyment discussed in the last section (music, play, festivity, sport, etc.), they are clearly a poor fit for sensory pleasures like the sweet taste of strawberry. And, in fact, both Csikszentmihalyi and Frederickson make a distinction between the kinds of positive experience targeted by their theories and basic varieties of sensory or bodily pleasure (e.g., see Fredrickson, 2009, p. 38).

I agree that some such distinction is warranted (see below for my version). But whenever we make distinctions between certain kinds of positive feeling we are begging the question of the nature of positive experience in general. Philosophers who reject the unity of valence (Solomon, 2007; Labukt, 2012) also beg the question of how such radically different kinds of feeling are identified as positive in the first place. To be fair, the question is also begged by scientists who propose that different varieties of pleasure and pain constitute a “common currency” that is essential to decision making (e.g., Cabanac, 2002; Leknes and Tracey, 2008; Barrett and Bliss-Moreau, 2009; Grabenhorst and Rolls, 2011; Berridge and Kringelbach, 2013). This proposal calls for a theory that explains how the enormous variety of positive and negative feelings belongs to a single affective continuum.

The larger point that I am making is that any attempt to explain a certain kind of pleasure or enjoyment—our enjoyment of imaginative culture being a central case in point—is entangled with unresolved questions about the nature of affect in general. In this respect, mechanistic and enrichment approaches are in the same boat. But the enrichment approach has significant advantages that indicate the possibility of its being developed into a more complete theory, one that can cover both “interactive” and “button-like” varieties of positive experience.

The first step toward a more complete theory is to refine our concept of the “richness of experience.” We need a concept that is general enough to apply to a wide variety of experiences and yet manages to illuminate the “intrinsically motivating and inherently good or bad” nature of all affective feelings. I suggest that the right kind of concept is supplied by philosopher Whitehead’s (1978) account of *intensity*.

According to Whitehead, all satisfaction consists in the *intensity* of feeling, which he defines as a relational feature constituted by *contrast*. For instance, think of the intense contrast of blue and yellow. Whitehead’s concept of contrast is much more general than this, however. It includes *all* the various distinct qualities and features belonging to all sensory modes and refers to *whatever overall effect is achieved by the interrelation of two or more qualities within a single feeling*.

Once contrast has been generalized in this way, every single conscious feeling can be described as a unitary complex contrast comprising an indefinite variety of qualities and features. Also, contrast in this more general sense refers not just to the accentuation of difference but to all the different ways in which diverse aspects of experience condition one another. For example, when we use lighting and music to enhance the ambience of a dinner party these elements may not literally contrast with the food and conversation—most of the time they are not even noticed—but insofar as they condition other aspects of experience they participate in the overall contrast of feeling. Contrast, in this sense, is a way of describing the relational nature of all qualities and, even more generally, the overall compositional character of experience. Accordingly, when Whitehead claims that satisfaction consists in the intensity of feeling, he is claiming that satisfaction consists in *richness of contrast*—the greater the contrast, the greater the satisfaction.

What exactly does it mean to *increase contrast* in this more generalized sense? Importantly, contrasts of feeling can vary in two ways: *strength* and *diversity*. Strength refers to the distinctness of the different components of feeling, while diversity refers to the number of distinctly discriminable components. As suggested by the way distinctness enters into both definitions, these two dimensions of contrast are interrelated: to count as a distinctly discriminable component of feeling requires a minimum level of strength. At the same time, these dimensions can vary somewhat independently: feelings may have high strength and low diversity (think of a black circle on a white background), or low strength and high diversity (think of the noise on a TV screen). In general, we find a trade-off between changes of strength and diversity: increases in one dimension are generally balanced by decreases in the other (think of how diversity changes as ingredients are added to a dish). It is possible, however, to have feelings marked by high levels of both strength and diversity of contrast.

Now that these two dimensions of contrast have been distinguished, we can define intensity, and thus richness of experience, with greater precision: *intensity is strength of contrast compounded by diversity*. On this view, the most inherently satisfying feelings are characterized by a diversity of strongly distinct qualities—think of the multicolored radiance of a stained-glass window or the richly textured sound of a jazz quintet. Notice that Whitehead’s intensity is not what we usually mean by an intense feeling (a sharp pain is not intense in the sense being described here). To prevent confusion, I prefer to refer to this feature as *harmonic intensity*. Whitehead’s intensity is “harmonic” because it has to with the way diverse elements of feeling condition one another as components of a single unified feeling.

Before moving on, it important to note both the advantages and the limitations of the enrichment approach as it has been elaborated so far. Let us start with the limitations.

Can we equate the positivity of all positive experience with harmonic intensity? Alas, no. Although we can confirm that *some* positive experiences are marked by harmonic intensity, it is easy to find a wide range of exceptions—strong pleasures that lack diversity (the taste of candy), soothing pleasures that lack strength (the sound of a noise machine), and so on. Moreover, even where harmonic intensity does show up, it does not *necessarily* constitute positive feeling: we can feel harmonic intensity without being pleased by it. And even if we can confirm that harmonic intensity is *generally* pleasing, in most situations it is not easily gauged. Certainly, when someone asks us how we feel, we do not examine the harmonic intensity of our experience. In conclusion, it seems that harmonic intensity, insofar as it can be discerned, cannot be identified with the positivity of feeling.

Nevertheless, it is important to recognize that harmonic intensity meets the criteria set forth at the end of the previous section. It is an “adverbial” feature that modifies experience across all sense modalities: it is evident that we *can* feel with more or less harmonic intensity; the harmonic intensity of experience *does* vary. Furthermore, *in some cases*, it is evident that this variation is inherently connected to the felt value of experience, that is, to the “inherently motivating and intrinsically good or bad” nature of affect (p. 246). These features indicate that harmonic intensity merits careful consideration.

Perhaps the best illustration of these two key features—the multi-modal nature of harmonic intensity and its inherent connection to value—is found in the popular animated film *Ratatouille* (Bird, 2007). The entire plot of this film revolves around the special culinary genius of the protagonist, a rat named Remy. To show this genius to the audience, in an early scene the film depicts Remy’s enjoyment of cheese and strawberry as a kind of synesthesia using swirls and throbs of colors and strands of music. First, as he tastes the cheese and strawberry separately, the two tastes are represented as distinct patterns of moving colors, each accompanied by a different musical theme. Then, when he tastes the cheese and strawberry together, the accompanying colors and music are joined together in a notably more lively, intensified form—with the result that we can *see* and *hear* the lively contrast made by the taste of cheese and strawberry. What makes this scene so revealing is the way it exploits our intuitive understanding of harmonic intensity as a multi-modal characteristic of sensory enjoyment. We intuitively grasp the connection between our experience of intensified music and color and Remy’s enjoyment of cheese and strawberry.^1^

Thus, within a limited range of experiences, the concept of harmonic intensity seems to capture something important about our experience of positive valence. At the very least, it seems to provide a clue about the kind of theory we are looking for.

## The Causal Enrichment of Experience

In this section I present the outlines of a theory of affect that extends the enrichment approach to the causal dynamics of experience. The argument now enters its most abstract phase, as consciousness is treated as a dynamical system. Also, insofar as it goes beyond the reach of current experimental evidence, this argument must be treated as speculative hypothesis. It is not without empirical basis, however: it draws from recent studies of the dynamics of consciousness and is intended to contribute to this research through the formulation of testable claims about the dynamic and energetic underpinnings of affect.

A variety of scientific approaches to the empirical investigation of consciousness have emerged during the past 25 years (for an overview, see Northoff and Lamme, 2020). This research is powered by huge amounts of data harvested from neuroimaging as well as various techniques for analyzing and modeling this data (e.g., Breakspear, 2017). For present purposes, all but the gist of this work must be left in the background. The general aim of these studies is to connect features of consciousness with dynamic variables at the “systemic molar level of neural activity” (Northoff and Lamme, 2020, p. 369). The basic guiding hypothesis behind this approach is the idea that consciousness has a distinctive “dynamic signature” that can be described and measured.

What do these studies hope to reveal about consciousness? How do they get around the so-called “hard problem”? Although the explanatory logic of this research has not been spelled out in detail, it seems to be guided by the expectation that dynamic descriptions of neural activity in conscious subjects will turn out to be isomorphic with experience, and that this isomorphism will tell us something about the nature of experience (or at least help us to refine our questions). As far as I can tell, none of these approaches has so far ventured to explain *why* certain levels or kinds of neural activity give rise to consciousness; for now, the goal is to describe and measure this activity as accurately as possible. Nevertheless, these approaches show that it is possible to make progress by observing how differences of certain measures of brain dynamics correspond to reportable differences of experience.

Many of these approaches seek to understand the difference between unconscious and conscious states in terms of some measure of the “complexity” of neural activity. Complexity can be defined and measured in many different ways, however, and scientists do not yet agree on how best to define and measure the complexity that specifically corresponds to consciousness. Nevertheless, as a basic orientation this “complexity thesis” seems to accord well with experience. Each of our conscious feelings is a richly structured, complex whole (Haun et al., 2017), and no two feelings are alike (Edelman and Tononi, 2000). It seems reasonable, then, to expect that the neural dynamics of consciousness can be distinguished by the following three features: (1) it must be integrated, (2) it must be richly structured, and (3) it must have access to an enormous repertoire of possible states. Different methods target these features differently. Integration can be measured by spatiotemporal coordination (Northoff and Huang, 2017), structure can be measured by “perturbational complexity” (Casali et al., 2013), and repertoire size can be measured by entropy (Carhart-Harris et al., 2014). All of these measurements can be used to distinguish conscious from unconscious states.

Here we are interested in the possibility that these same kinds of measurements can also be used to distinguish between basic levels or kinds of experience, such as levels of arousal (sleepy vs. awake) and, especially, changes of affective valence (positive vs. negative). As I will discuss momentarily, some lines of research are actively exploring this possibility, although to my knowledge none has tried to describe and measure the dynamic signature of affect. In what follows I move toward a dynamical characterization of affect that is most closely related to the third feature listed above: the size of the dynamic repertoire within which conscious feeling is determined. We begin with a brief discussion of the concept of *dynamic repertoire* and another, closely related concept, the *differentiation* of conscious states.

The *dynamic repertoire* of a system is the set of states that can be presently accessed by that system (Hadriche et al., 2013, p. 449; Hudetz et al., 2015). The dynamic repertoire of a system is not the same as its state space, that is, the total set of all possible states defined by all possible values of its component variables. A dynamic repertoire is a lower dimensional subset of the state space: it describes how the state space is partitioned into qualitatively distinct *macrostates* according to differences of stability and thus, in some cases, can be represented by an “attractor landscape.” So, for instance, a noisy system with a large state space may have very few stably differentiable macrostates and therefore a small dynamic repertoire. As discussed below, a critical feature of dynamic repertoires is that they can change as different states are stabilized or destabilized.

*Differentiation* has to do with the way in which states of a complex system are causally determined in relation to other states in the dynamic repertoire of the system. Notice that differentiation is a *causal concept*, although it is frequently associated with certain dynamical and informational features. In particular, according to a number of theories, conscious brain states are distinguished by their high differentiation (e.g., Edelman and Tononi, 2000; Hudetz et al., 2015; Carhart-Harris, 2018). Importantly, for a state to be highly differentiated it has to belong to a causally integrated system with a large repertoire of possible states—concepts of differentiation and dynamic repertoire are thus closely related.

For example, consider the countless different images that can be produced by a digital camera (Tononi, 2008). Regardless of how they might appear to us, these images do not constitute a dynamic repertoire and thus should not be thought of as highly differentiated. The reason has to do with the camera’s lack of causal integration. Because the sensors of the camera’s light-sensitive array are activated independently of one another, each image produced by this array is not causally differentiated from other possible images. Rather each image is an aggregate of the various independently determined sensor states, each of which is minimally differentiated.

Insofar as causal integration can be established or assumed—by no means a trivial matter—measures of the dynamic repertoire of brain activity can serve as an index of the differentiation of “whole-brain” states. The key supposition of the following discussion is that *changes of the dynamic repertoire are directly related to changes of differentiation*.

Investigations of the dynamic repertoire of whole-brain activity focus primarily on estimating its size, or number of states.^2^ For instance, one study found that the transition from unconscious to conscious states corresponds to an increase in repertoire size (Hudetz et al., 2015). This finding seems to confirm the view that conscious states are distinguished by high levels of differentiation. Other studies have found that psychedelic states induced by psilocybin are also linked to increases in repertoire size (Carhart-Harris et al., 2014; Tagliazucchi et al., 2014). This finding suggests that conscious states can be *more or less differentiated*. Moreover, it suggests a kind of “causal enrichment” could be defined in relation to the differentiation of conscious states. Could it be that differentiation is what determines affective valence?

In fact, scientists working in this line of research sometimes use the term *richness* in reference to the size of the conscious dynamic repertoire (e.g., Hudetz et al., 2015; Carhart-Harris, 2018; Deco et al., 2019). This usage seems intended to suggest that changes in the size or richness of the repertoire, and thus changes of differentiation, are related to changes of experience. But *what does it feel like when the dynamic repertoire expands or contracts?* What is the difference from a first-person perspective between a highly differentiated state and a less differentiated (but still conscious) state?

In the experiments with psilocybin, changes of repertoire are indexed by changes of entropy and are associated with the highly flexible, hyper-associative, or “divergent” style of thinking commonly observed in subjects undergoing psychedelic experiences (Carhart-Harris et al., 2014 Tagliazucchi et al., 2014). Neuroscientist Carhart-Harris (2018, p. 169) concludes from these studies that expansion of the repertoire corresponds to increased “richness” of conscious contents as well as cognitive flexibility. However, although Carthart-Harris’s hypothesis that entropy marks a “key property” of consciousness is exactly the kind of dynamic connection we are seeking, what he calls richness is *not* valence. As Carthart-Harris himself points out, entropy and its correlated features—greater content and cognitive flexibility—are not necessarily markers of optimal experience, as their increase is accompanied by decreases of other features and reaches an upper limit beyond which consciousness is lost.

What might the dynamic signature of valence be, then, if not repertoire size? Carthart-Harris’s entropic hypothesis points toward a different kind of richness that is implied by the concept of dynamical repertoire. When defined as uncertainty, entropy measures only the size or diversity of the dynamical repertoire: the larger the repertoire, the greater the uncertainty of its states, the greater the entropy. It must be remembered, however, that all changes of the dynamic repertoire are *changes of stability* (bifurcations). The dynamic repertoire is not the total state space but rather a lower dimensional set of *stably differentiable* macrostates. For example, the dynamic repertoire of the human body does not include every possible configuration but only relatively stable states (including patterns of movement), such as sitting, standing, walking, and running. The differentiation of these four states depends on their relative stability, and it is evident that their stability can change. When we are tired, sitting becomes more stable relative to standing, walking, and running, but as long as the latter states can be accessed and maintained they still belong to our dynamic repertoire. Thus, changes of stability allow for the dynamic repertoire of a system to change in more than one way: it can change in respect of the number of states, and also in respect of the *relative stability of these states*. These changes are closely related—*they are both changes of stability*—but they are not quite the same.

This point can be clarified by thinking of a changing dynamic repertoire as an attractor landscape: a surface whose peaks and valleys correspond to different levels of stability, so that each valley represents a differentiable state of the repertoire. So, for example, the single-state repertoire of a monostable system is represented by a single large valley, while the two states of bistable system are represented by two valleys separated by a peak of instability. As the repertoire increases in size, the landscape begins to resemble rugged mountains or gently rolling hills, depending on differences of stability. And this is the key point: repertoires of the same size, that is, with the same *number* of states, can be very different in another respect—rugged or smooth—depending on relative differences of stability.

The repertoires of conscious neural systems are generally understood to be characterized by *metastability*, which means that there are no true stable states, only more or less differentiable trajectories through state space (Kelso, 2012). We can think of a metastable repertoire as a gently undulating surface in which a rolling marble would meander about, lingering in various places but never coming to rest. Although differences of stability approach a minimum in these systems, they must still be present enough for different states of a dynamic repertoire to be identified as such. Moreover, I suggest that even in the metastable regime there can be significant differences of relative stability. This possibility is implied by the increased cognitive flexibility—the ease and rapidity of transition between states—associated with expanded repertoires (Carhart-Harris, 2018).

Drawing out the implicit feature of relative stability gives us a more complex notion of the richness of the dynamic repertoire of consciousness and the differentiation of conscious states. Insofar as a system can vary in both the number and stability of its states, we can think of the richness of its dynamic repertoire in terms of these two basic variables. A rich repertoire, in this modified sense, is one that has a large number of relatively stable states: *richness is a function of both size and relative stability of the dynamic repertoire*. Again, in the case of consciousness, “relative” is a critical qualification, as we are talking about metastable repertoires in which stability approaches a minimum. Nevertheless, insofar as the relative stability of conscious states can vary, states can be more or less differentiated in this respect. To distinguish this kind of differentiation from that which depends only on the size of the repertoire, I call it the *differentiated-ness* of conscious states.^3^

We can now formulate a more precise definition of affect as the causal enrichment of experience. I propose that valence is determined by the differentiated-ness of conscious states, so that positive feelings are caused by an increase of differentiated-ness and negative feelings are caused by its decrease. In other words, I propose that a good feeling is a highly differentiated feeling and a bad feeling is a poorly differentiated feeling.

Although this move builds upon the preceding arguments, it makes an inferential leap to a hypothesis of the relationship between affective valence and the dynamics of conscious experience. The strongest support for this claim comes from drawing out its implications, some of which will be sketched below. Nevertheless, it is important to see how this hypothesis is based on an intuitive connection between differentiated-ness and harmonic intensity.

Recall that harmonic intensity was defined in the last section as a basic “adverbial” feature of experience related to the qualitative richness of experience. Specifically, harmonic intensity is strength of contrast compounded by diversity. Differentiated-ness bears a certain resemblance to harmonic intensity insofar as it also varies in two interrelated dimensions—relative stability and size of the dynamic repertoire. Moreover, if stability corresponds to strength, we can say that differentiated-ness is strength of differentiation compounded by diversity. Based on this resemblance, we can infer that greater differentiated-ness yields something like the harmonic intensity we find in the qualitative contrasts of feeling.

The intuitive basis of my proposal, then, is the “harmonic intuition” that our feeling of value is determined by a harmonic feature of experience. This intuition is based, in turn, on the inherent appeal of feelings marked by harmonic intensity. But as I have pointed out above, valence cannot be explained by the *explicit* harmonic intensity that can be detected in the contents of feeling. Instead, I am now proposing that valence is determined by an *implicit* harmonic feature related to the causal determination of these contents. One could say that differentiated-ness refers to the way in which feelings are “harmonically individuated” within the dynamic repertoire of consciousness. Moreover, my thesis claims that changes of differentiated-ness somehow modifies experience by adding to (or subtracting from) its overall harmonic intensity even though it is never revealed to us as an explicit, discernible feature. Indeed, the differentiated-ness of feeling *cannot* be revealed as an explicit feature, as it belongs to the way in which all contents of feeling are determined.

Admittedly, even if space were not limited, such abstract arguments can only go so far. Again, the best way to evaluate this harmonic theory of affect is to draw out its implications for experience and relevant areas of scientific research. In the next section, I sketch some of the implications of this thesis for our understanding of imaginative culture. In the remainder of this section, I want to show how the harmonic theory can be used to distinguish between different kinds of positive experience and to understand these as belonging to a single, unified affective continuum.

First, the harmonic theory makes a distinction between two ways in which experience can be enriched: richness of content (harmonic intensity), and richness of causal determination (differentiated-ness). These two senses of richness are probably not unrelated; in general, we can expect that feelings with rich contents are also richly differentiated. With this distinction, the theory of causal enrichment allows for a much greater variety of feelings to count as enriched feelings, and this is a significant advance for the enrichment approach. For example, feelings dominated by a single quality—the vivid blue of a clear sky, the intense flavor of dark chocolate, the warm timber of a cello, the lush feel of velvet—can all be enriched feelings if they are strongly differentiated from a diverse range of other feelings within the conscious repertoire.

**Figure 1:**
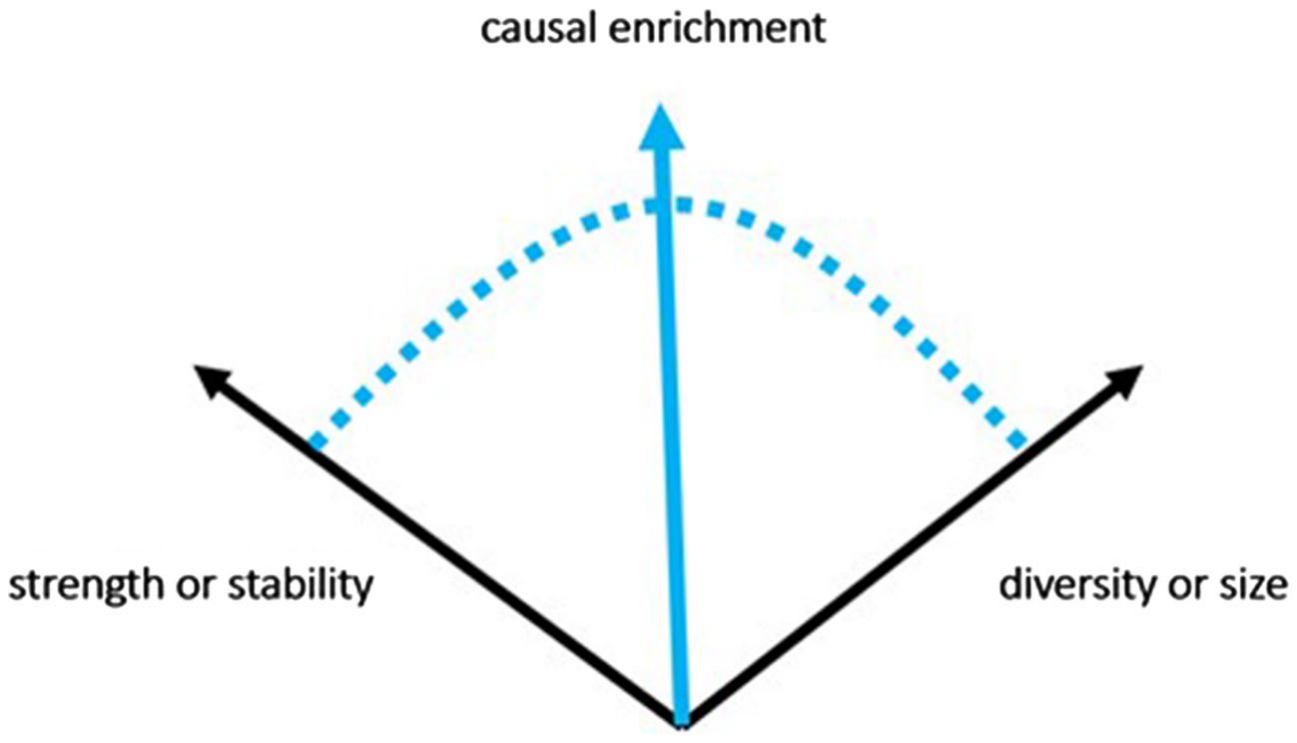
Causal enrichment can be increased by adding stability (strength), size (diversity), or both.

Second, because richness is a function of two dimensions—strength and diversity—it is possible for feelings to be enriched in more than one way, as indicated above (Figure 1).

The dimensionality of richness allows for different varieties of both positive and negative tone, as valence depends only on the overall richness of differentiation or, more precisely, on overall strength of differentiation compounded by diversity. Following Whitehead (1978), I suggest that feelings marked by increased diversity have a *wide* tone, while feelings marked by increased strength have a *narrow* tone. For example, an intense sensory pleasure has a narrow tone, while a pleasantly relaxing feeling has a wide tone. This variability of tone is more or less congruent with other models of affect (*cf*. Barrett and Bliss-Moreau, 2009).

A third distinction depends on the way in which the energy required for causal enrichment is obtained. This point requires some elaboration.

In general, the differentiation of macrostates in complex, non-equilibrium systems constitutes a kind of order and thus requires a constant supply of energy. The dynamic stable states of whirlpools, Rayleigh–Benárd cells, and other “dissipative structures” (Kondepudi et al., 2020) are common examples of this connection between stability and energy flow in non-equilibrium systems.^4^ Causal enrichment requires an increase of the stability or number of states in the dynamic repertoire (or both), and thus requires more energy.

In the case of the conscious brain, the states to which we are referring are *metastable* patterns of spatiotemporal correlation within a context of continuous intrinsic neural activity that—like a whirlpool—depends on a constant flow of energy. This means that the conscious dynamic repertoire, as well as each and every state that is differentiated within this repertoire, can be thought of as a more or less ordered flow of energy, that is, as a dissipative structure. Any change of the conscious dynamic repertoire should correspond to a change in the way that energy flows through associated patterns of neural activity and should in principle be measurable as such.

A key implication of the harmonic theory, then, is that the causal enrichment required for positive experience of any kind depends in part on increased energy use. How does this square with what we know about the energetics of the brain?

It is frequently observed that the brain is an “expensive tissue” that accounts for over 20% of the body’s energy consumption despite constituting only 2% of its mass (Campbell, 2010). Also, the rate at which the brain uses energy changes, and the brain seems to be capable of regulating its own energy use (*ibid*.). More specifically, there is ample evidence that the brain’s energy use varies both temporally and spatially in connection with neural activity (Watts et al., 2018), which, in turn, is widely believed to vary in response to task-specific cognitive demands. In addition, some have suggested that the transition to consciousness is connected with greater energy use (Shulman et al., 2009). Thus, the idea that changes of a basic property of experience are connected to changes of energy use fits well with our current picture of the energetics of the brain.

However, in a recent article that explores the energetic nature of consciousness, Pepperell (2018, p. 3) cautions that there is “no clear correlation between total amount of energy used by the brain…and the level of consciousness detectable in the person”. Instead, he proposes that levels of consciousness correspond to the “*organization* of energetic activity” in the brain, such that “wakeful conscious states are associated with *more complex organization*” (*ibid*., italics added). Similarly, a recent study of the relation between consciousness and the spatiotemporal coordination of neural activity claims that it is the “degree of fractal and scale-free organization” rather than “mere level of global activity or metabolism itself that is central for the level or state of consciousness” (Northoff and Huang, 2017, p. 634).

Nevertheless, insofar as complex organization entails greater energy use, these perspectives suggest that an increased supply of energy is a necessary condition for causal enrichment. Moreover, the harmonic theory suggests that causal enrichment can take different forms depending on how this condition is met. A broad distinction can be drawn between two kinds of energy increase: those that simply add energy to the system, resulting in increased strength, and those that add both energy and organization, resulting in both increased strength and diversity. Insofar as both lead to an overall increase of differentiated-ness, both can lead to increased positivity.

Furthermore, I suggest that these two kinds of energy increase correspond to the two basic kinds of positive experience discussed in earlier sections: the “button-like” pleasure associated with “hard-wired” affective responses and the “interactive” enjoyment found in imaginative culture, play, and wide variety of other activities. I will refer hereafter to these two kinds of positive experience as *pleasure* and *enjoyment*. In general, pleasure and enjoyment are characterized by different kinds of affective tone: pleasure is the result of added strength and so usually has a narrow tone, while enjoyment is the result of increased strength and diversity, and so is usually marked by the explicit richness of harmonic intensity.^5^

Pleasure and enjoyment also differ in the kinds of energy sources that they require. Intuitively, it should be relatively easy for the body to give itself an energetic “boost” that momentarily strengthens feeling. We do not know how this is accomplished in the case of pleasure, but the brain’s constant regulation of its own energy use suggests that it is possible. Meanwhile, for the kind of enrichment that supports enjoyment, it may be necessary for experience to be interactively coupled with a richly structured source of energy. Accordingly, I suggest that pleasure and enjoyment correspond to two sources of causal enrichment: (1) specially adapted regulatory mechanisms of the body and brain (neurochemicals and the like), and (2) engagement with richly structured “ambient arrays” of energy described by the ecological theory of perceptual experience (Gibson, 1986).

For readers who are unfamiliar with ecological psychology this last point will seem to come from left field. Unfortunately, the details of this approach to perception cannot be reviewed here. Let it suffice to say that the harmonic theory of enjoyment can draw upon the ecological view of perception as an interactive process that quite literally “feeds off” the richly structured gradients of light, sound, and chemicals carried by the media of air and water (*ibid*., p. 16–17). Normally ecological psychology focuses on the way in which these energy gradients serve as sources of information about things in our environment and the kinds of activities that they afford (p. 19–44). But within the ecological framework, it is clear that the thermodynamic nature of perception is not fundamentally different in kind from other organic processes (see, especially, Swenson and Turvey, 1991). In many if not most contexts of animal life, perceptual engagement with ambient energy is driven by a search for meanings relevant to current needs and interests, not for the sake of causal enrichment *per se*. Nevertheless, the preceding claims about the energetic nature of enjoyment, when combined with the ecological perspective, suggest that the capacity for enrichment—and the pursuit of this enrichment for its own sake—is intrinsic to perceptual experience.

It follows that enjoyment is a basic and universal dimension of animal life, an intensified form of experience that arises spontaneously whenever conditions allow. Probably the most commonly observed expression of this universal tendency is play behavior, which, according to some biologists, can be found in all or nearly all forms of animate life (Burghardt, 2005). In general terms, the conditions for enjoyment are enhanced versions of the same ecological conditions that hold for experience in general: engagement with richly structured gradients of ambient energy, which in turn depend on how light and sound interact with substances, surfaces, and events in the surrounding environment. In other words, the harmonic theory suggests that, unlike the capacity for pleasure, whose specialized mechanisms are products of natural selection, the capacity for enjoyment is not an adaptation. Rather, enjoyment is inherent to experience: any species capable of experience will ipso facto be capable of enjoyment. More species-specific forms of enjoyment depend on more specific capacities of perceptual engagement.

## Imaginative Culture and the Enjoyment of Enriched Meaning

We come at last to imaginative culture. In light of previous discussion, we can affirm that our experience of imaginative culture is a kind of enjoyment, that is, an interactive form of positive experience whose causal enrichment depends on engagement with richly structured sources of ambient energy. But it is evidently much more than this. Unlike play, which seems to be very widespread, enjoyment of imaginative culture seems to be uniquely human. Also, it differs from other kinds of human enjoyment. I suggest that the most distinguishing feature of our experience of imaginative culture is its meaningfulness. In this final section, I will be focusing on imaginative culture as a source of enriched meaning, and I will be focusing on music as representative example.

Recall that imaginative culture was defined at the outset as the use of specially prepared media for the purpose of enriched experience. We are not the only species capable of altering our environment for the purpose of enjoyment (think of otters and their slides). Imaginative culture can be further distinguished by its highly distinctive ways of *organizing* materials and activities for purposes of enjoyment—in the case of music and dance, patterns of sound, and coordinated movements. But again, a number of animals engage in similar activities to attract the attention of conspecifics. I suggest that what most distinguishes imaginative culture is the way in which meaning is perceived in and expressed through its various media.

This feature seems to be grounded in the flexibility with which we can switch between different ways of perceiving. Our understanding of this flexibility depends on how we understand the perception of meaning in general. On the ecological view (Gibson, 1986; Clarke, 2005), meaning is directly perceived, not something added to “sense data” by layers of “information processing.” But this is not to say that it is the same for everyone. Rather, meaning is direct in the sense that it is *specified* by perceptual engagement with the ambient energy that informs perception. *What* meaning is specified depends on factors on both sides of engagement, including the way in which perceptual engagement is intentionally directed.

So, for example, when we listen to music, the meaning we hear is not just a result of how musical sounds are specially produced and organized: we listen to these sounds in a particular way, and as a result, we hear their meaning differently from the way we hear the meaning of everyday sounds. Indeed, the same sound (from a physical standpoint) can be heard in different ways depending on whether it is heard as music. Think of how the sound of someone striking wood changes depending on whether we hear it as a knock at the door or as musical rhythm. I suggest that the difference can be roughly described as follows: With everyday sounds, we direct our attention to their physical source, while with musical sounds we direct our attention to how they are related to one another. This is far from a complete account of musical meaning, of course. But it should be acknowledged that such an account is in any case unavailable, as we do not yet know how all the different meanings that we hear in music are specified. In particular, we still do not have a well-established scientific explanation of the enormous range and subtlety of emotion that we can hear in music.

We seem, then, to have reached an impasse. After all that has been said about affect, it turns out that our understanding of the enjoyment of imaginative culture depends, at least in part, on our understanding of the experience of meaning.^6^ And the problem of meaning is as important and as challenging as the problem of affect. Fortunately, the ecological thesis that meaning is directly specified by perceptual activity allows us to finesse the problem of meaning. We need not describe the dynamic variables that specify particular kinds of meaning in order to consider the implications of direct specification for enjoyment. What matters most for present discussion is the *richness* of meaning specified by imaginative culture. The primary case in point is our experience of meaning in music.

Perhaps because music is so widely enjoyed, it is easy to overlook the fact that it offers direct experiential access to an extraordinary range, depth, and subtlety of meaning. The meaningfulness of music would be an astounding discovery if it were not already such a commonplace feature of human life. Among the many different kinds of meaning experienced in music, perhaps the most common, and also the most powerful, is emotion. Moreover, one of the most striking features of our experience of emotion in music is its extraordinarily nuanced character.

Consider how pains of the heart are differently expressed by popular musical traditions—how each tradition uses a different emotional palette. The emotional nuances of blues are different from those of flamenco, and so on. Or consider the different nuances brought out by great performances of the same song—say, “One More for My Baby (and One More for the Road)” as sung by Holiday, Sinatra, Bennett, and Fitzgerald. It is amazing to consider the nuance of feeling that can be found on the same street of the same emotional neighborhood. There are no words to describe such fine differences. But they are not trivial: our appreciation of these nuances and our enjoyment of music go hand in hand. Whenever we enjoy the emotion of a particular song, what we relish is not just the experience of emotion *per se* but *this* unique feeling of emotion brought forth by *this* performance of this song.

The point that I am making is that the *sine qua non* of musical enjoyment—as made manifest by our enjoyment of emotion—is the extraordinary differentiated-ness of our experience of musical meaning. As famously claimed by Mendelssohn-Bartholdy (2019), the feelings of music are too fine (*bestimmte*) for words to describe (*cf*. Barrett and Schulkin, 2017). This view of musical enjoyment is not uncommon (Langer, 1996, p. 222–245), but neither is it self-evident. The elaboration of this view into a comprehensive thesis about our enjoyment of imaginative culture is even more tenuous. For present purposes, however, my intention is not to defend these positions but to show how they fit together within a theoretical framework that can help us to understand our fascination with imaginative culture.

The theory presented in this article is able to confirm Dewey’s claim that imaginative culture is driven in large part by our “preoccupation with direct enjoyment.” Moreover, it is able to describe in general terms how our enjoyment of imaginative culture relates to other kinds of enjoyment and pleasure. The thesis that our enjoyment of imaginative culture is based in experience of enriched meaning points to other motives besides enjoyment, however. In particular, the enormous popularity of songs about heartbreak suggests that imaginative culture serves an additional, albeit closely related, purpose. In short, if music and other forms of imaginative culture are just for enjoyment, why do we so often use them to explore the darkest and most difficult aspects of human life?

The question that I am raising is a version of the classic “paradox of tragedy.” In general terms, this paradox has to do with our tendency to use imaginative culture to cultivate enriched experiences of emotionally troubling events that in normal life we try to avoid. Most explanations of this paradox belong to two types (*cf*. Levinson, 2014). According to one type, imaginative culture gives us the opportunity to experience these events “at a distance,” that is, without actually undergoing the troubling emotions that normally accompany them. According to another type, imaginative culture *does* induce troubling emotions, but it does so in a special way that is cathartic. Without ruling these out, the harmonic theory of affect suggests a third possibility. Through the enrichment of meaning, imaginative culture gives us the opportunity to transfigure our experience of these events, at least momentarily, in ways that are deeply gratifying and life-affirming.

An important part of this transfiguration is a change of affective tone. As evidenced by the popularity of songs about heartbreak, a musically enriched experience of heartbreak is deeply satisfying in a way that our normal experience of heartbreak is not. The harmonic theory explains this transfiguration by its claim that positive valence *just is* the enrichment of experience. Therefore, though it may seem paradoxical, an enriched experience of tragedy will feel better simply by virtue of its richness. A corollary of this thesis is that artistic depictions of tragic events are not satisfying except insofar as they achieve this richness, which helps to explain why art fails when it simply tries to “push our buttons.” It is not easy to produce an enriched experience of tragedy.

At the same time, the harmonic theory indicates that the transfiguration of experience wrought by imaginative culture is much more than a change of affective tone. An enriched experience of tragedy is also a cognitive achievement, which is why profound enjoyment of the arts is so often accompanied by an enlarged and refined experience of life as a whole. The harmonic theory also accounts for this contribution of imaginative culture, as it ties the affective enrichment of experience to the refinement of our conscious repertoire. It can even be said that imaginative culture serves to improve our capacity to feel. Often this improvement is fleeting—our insight into the beauty of life evaporates almost as soon as the music is over. But for many people, a regular diet of imaginative enjoyment is maintained not only for its own sake but also for the sake of cultivating an enriched capacity for everyday experience.

In summary, imaginative culture is critical to our understanding of affect in at least three ways. First, it makes manifest the enriched nature of enjoyment and positive experience in general. Second, its use of specially prepared media exemplifies the dependence of enjoyment on engagement with a richly structured environment. Third, the use of imaginative enrichment to transfigure our experience of the most troubling aspects of life affirms that positivity is not a special quality added to feeling but an improvement in our overall capacity to feel.

## Data Availability Statement

The original contributions presented in the study are included in the article/supplementary material, and further inquiries can be directed to the corresponding author.

## Author Contributions

The author confirms being the sole contributor of this work and has approved it for publication.

## Conflict of Interest

The author declares that the research was conducted in the absence of any commercial or financial relationships that could be construed as a potential conflict of interest.

## Publisher’s Note

All claims expressed in this article are solely those of the authors and do not necessarily represent those of their affiliated organizations, or those of the publisher, the editors and the reviewers. Any product that may be evaluated in this article, or claim that may be made by its manufacturer, is not guaranteed or endorsed by the publisher.

## Footnotes

^1^To create the synesthetic tasting sequence, Pixar specially commissioned the Canadian animator Michel Gagné; the sequence can be viewed on his YouTube channel and is highly recommended.

^2^One of many issues left in the background of this discussion is the question of how to distinguish the states of brain-wide activity. See Deco et al. (2019).

^3^Unlike differentiation, differentiated-ness is not measured by entropy or any other index of the sheer size of the dynamic repertoire of a system. Among the many different techniques for measuring and analyzing brain activity, I am not sure which would work best as an index of differentiated-ness.

^4^Notice that the relationship between stability and energy use in nonequilibrium or dissipative systems goes against our commonsense intuition that the most stable states are those with the least energy (like a marble that has settled into the bottom of a bowl).

^5^More precisely, the condition for enjoyment is a continuous flow of highly differentiated feelings (“implicit richness”). So, for instance, it is possible for us to find enjoyment in minimalist art (e.g., Rothko’s multiform paintings) or an austere Japanese rock garden insofar as these support a continuous flow of richly differentiated feeling.

^6^It should be clarified that meaning is being discussed here in a very broad sense that includes much more than representational content. Much of imaginative culture, ancient and modern, is “non-representational.” Nevertheless, all imaginative culture is experienced as having at least the kind of meaning that belongs to its particular medium. For example, the notes of a melody are heard as meaningfully related to one another, regardless of whatever other meaning they might have.
